# Supplementary material for: Virtual navigation tested on a mobile app is predictive of real-world wayfinding navigation performance
Source: PLoS One. 2019 Mar 18;14(3):e0213272. doi: 10.1371/journal.pone.0213272 (PMC6422266; doi:10.1371/journal.pone.0213272)
Supplement: S1 Fig — Maps of wayfinding Sea Hero Quest levels 1, 6, 11, 16 and 43. Starting position and facing direction are indicated by a pale blue arrow, ordered goals by red flags. Participants must memorize the map, and then navigate towards the goals in the right order as quick as possible. (PDF) [file pone.0213272.s001.pdf]

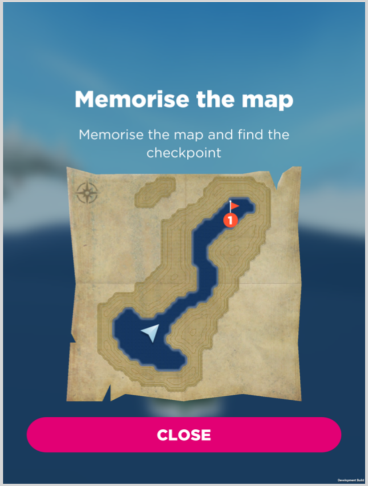
Memorise the map
Memorise the map and find the checkpoint
CLOSE

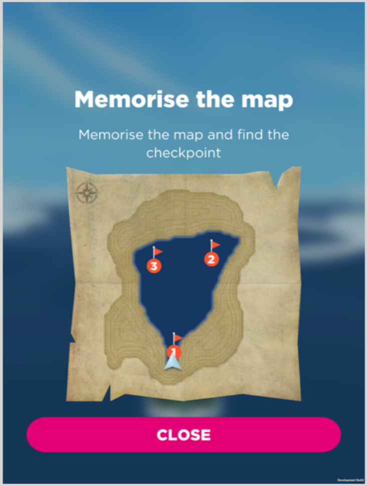
Memorise the map
Memorise the map and find the checkpoint
CLOSE

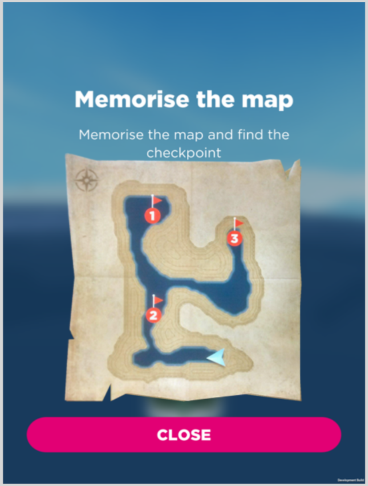
Memorise the map
Memorise the map and find the checkpoint
CLOSE

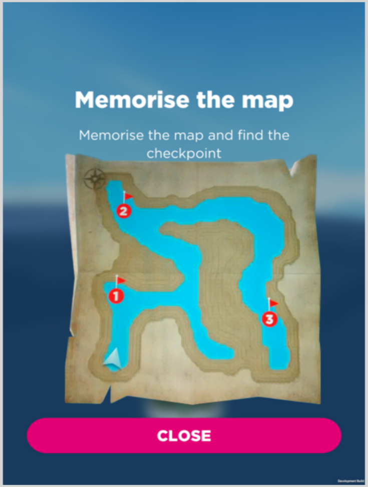
Memorise the map
Memorise the map and find the checkpoint
CLOSE

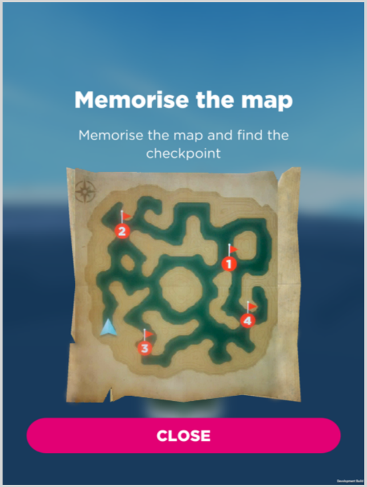
Memorise the map
Memorise the map and find the checkpoint
CLOSE
