# Supplementary figures and images for: Virtual navigation tested on a mobile app is predictive of real-world wayfinding navigation performance
Source: PLoS One. 2019 Mar 18;14(3):e0213272. doi: 10.1371/journal.pone.0213272 (PMC6422266; doi:10.1371/journal.pone.0213272)

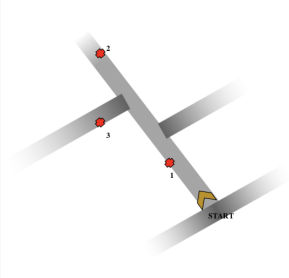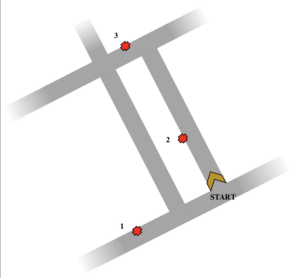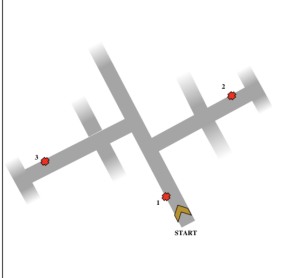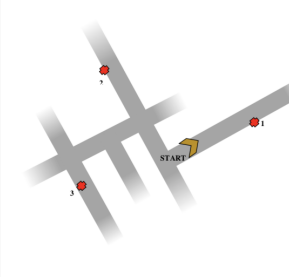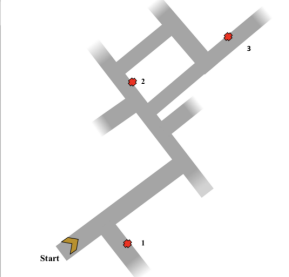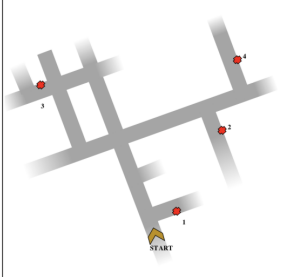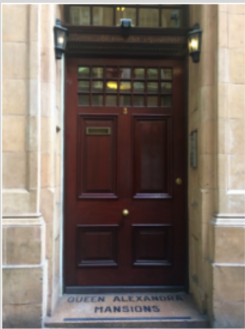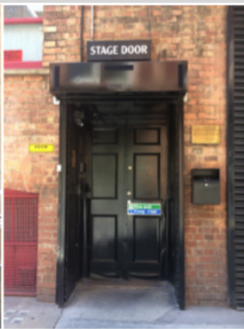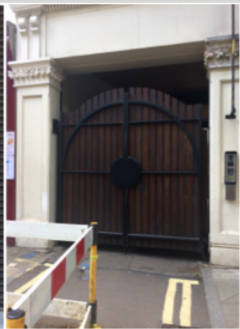

Supplement: S2 Fig — Maps of real-world wayfinding routes (top). Starting position and facing direction are indicated by a yellow arrow, ordered goals by red dots. Participants must memorize the map, and then walk towards the goals in the right order as quick as possible. Goals are materialized by remarkable doors (bottom). (PDF) [file pone.0213272.s002.pdf]

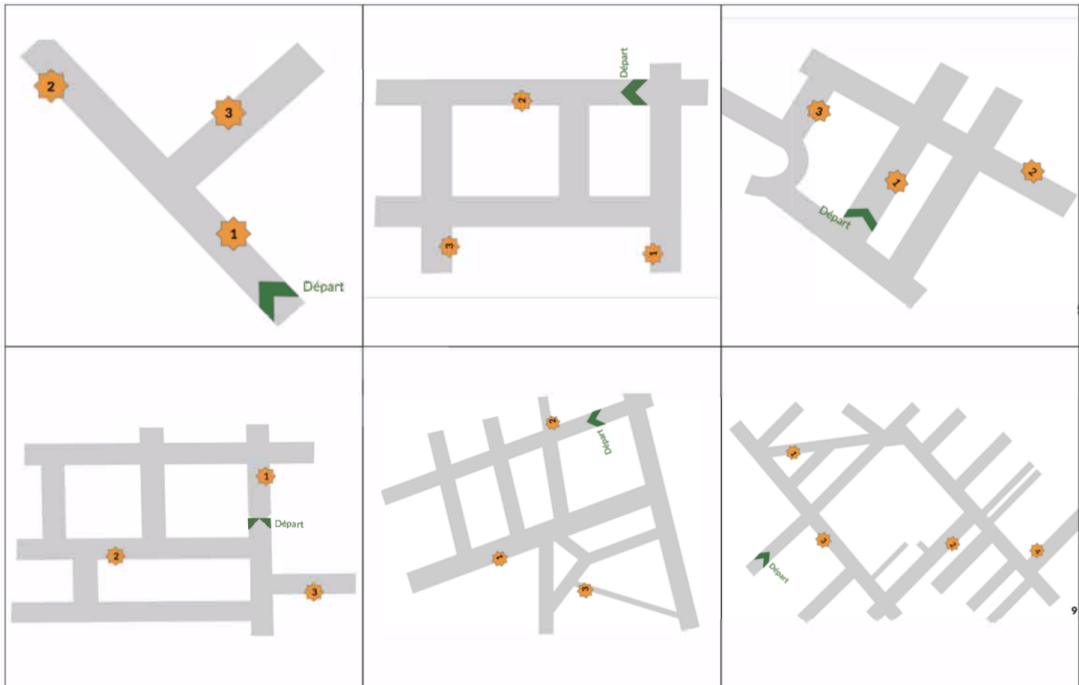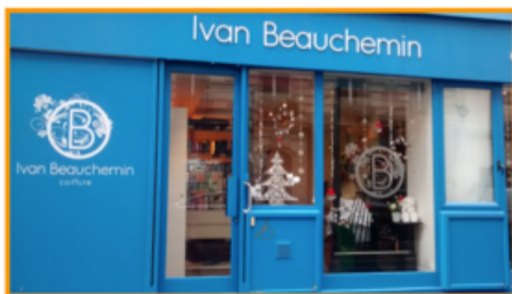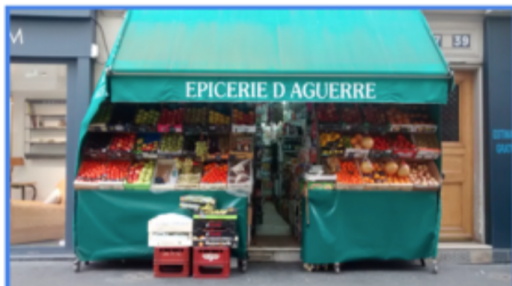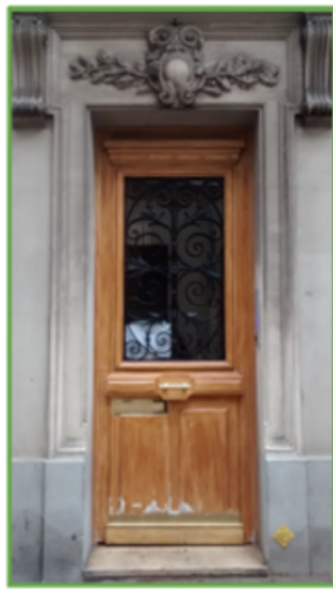

Supplement: S3 Fig — Maps of real-world wayfinding routes (top). Starting position and facing direction are indicated by a green arrow, ordered goals by yellow dots. Participants must memorize the map, and then walk towards the goals in the right order as quick as possible. Goals are materialized by remarkable facade (bottom). (PDF) [file pone.0213272.s003.pdf]

Level 74: five turns

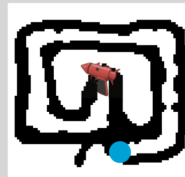

flare

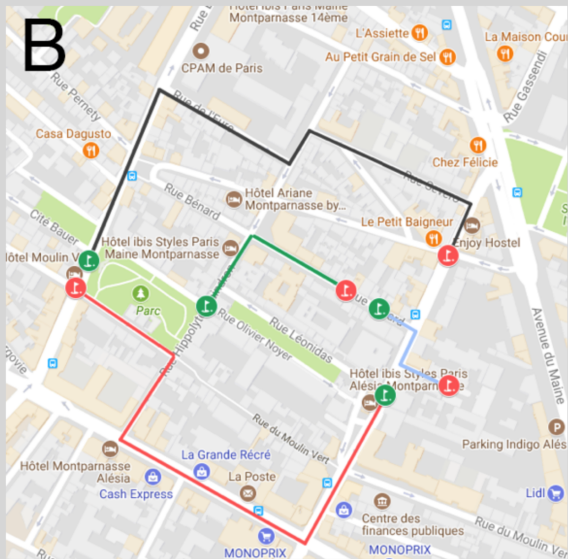

Supplement: S4 Fig — A—Maps of Sea Hero Quest path integration levels 14, 34, 44, 54 and 74. B—Itineraries of the path integration task in Paris (France). Each color corresponds to a different itinerary. (PDF) [file pone.0213272.s004.pdf]

Path Integration Performance (scaled)

2  
1.5  
1  
0.5  
0  
-0.5  
-1  
-1.5  
-2  
-2.5

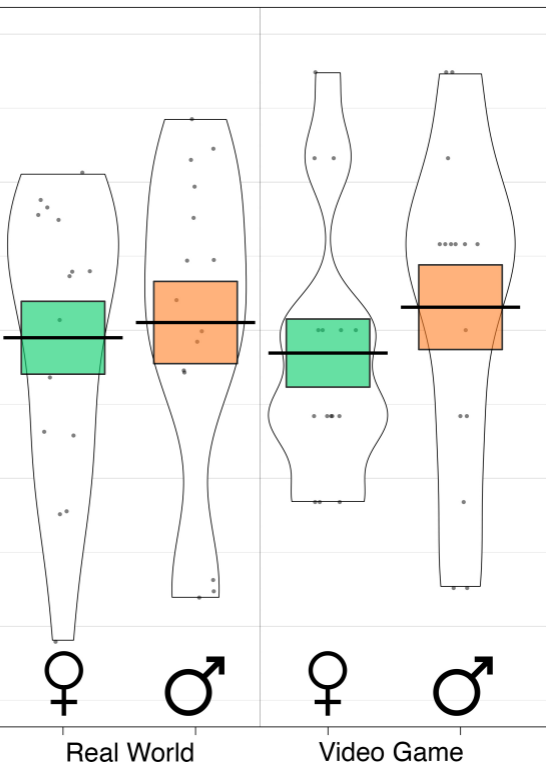

Supplement: S5 Fig — Gender differences for the path integration task in the video game (right) and in the real world (left) in Paris. In the real world, path integration performance is the opposite of the average error angle. In the video game, path integration performance is the average number of stars. Both measures have been standardized for comparison. Black dots represent individual data points. Error bars represent standard errors. (PDF) [file pone.0213272.s005.pdf]
